# Reopacification of posterior capsular opening after ND: YAG capsulotomy: 2 cases with the different presentation

**Published:** 2019

**Authors:** Subhash Joshi Rajesh

**Affiliations:** * Department of Ophthalmology, Vasantrao Naik Government Medical, College, Maharashtra, India

**Keywords:** posterior capsular opacification, ND: YAG laser capsulotomy, reopacification of the visual axis, Elschnig pearls, posterior capsulorhexis

## Abstract

**Purpose:** To report the complete opacification of posterior capsulotomy opening after neodymium: yttrium-aluminium-garnet (ND: YAG) posterior capsulotomy for posterior capsular opacification.

**Methods:** A 70-year-old female had uneventful cataract surgery by phacoemulsification technique with implantation of in the bag hydrophilic intraocular lens (IOL) in her left eye. Eight months after surgery, the patient developed posterior capsular opacification (PCO) for which ND: YAG capsulotomy was performed. However, she returned after 7 months first post ND: YAG capsulotomy with the blurring of vision and glare at night. Slit lamp examination revealed pearl like opacification of posterior capsule occupying the YAG capsulotomy opening. A repeated ND: YAG capsulotomy restored her vision to 20/ 20. Another patient operated for posterior polar cataract had dehiscence in the posterior capsule during cataract surgery. The patient was implanted with hydrophobic IOL in the capsular bag without performing an optic capture. The patient presented 3 years later with occlusion of the posterior capsular opening with mixed pearl-like and fibrotic PCO.

**Conclusion:** Occlusion of the posterior capsular opening may occur after ND: YAG capsulotomy and may cause a reduction in visual acuity. Pearl-like opacification is common to occur in reopacification of posterior capsular opening.

## Introduction

Posterior capsular opacification (PCO) is a common complication after extracapsular cataract surgery, which leads to blurring of vision. About 50% of the patients undergoing cataract surgery need capsulotomy after 3 years of cataract surgery [**1**]. Neodymium: yttrium-aluminium-garnet (ND: YAG) posterior capsulotomy restores lost vision. The procedure is simple, quick and causes rapid recovery of vision. YAG capsulotomy is a one-time procedure and rarely needs repetition. Recurrence of PCO is infrequent after YAG – capsulotomy. Recurrence can occur after cataract surgery in post-vitrectomized eyes and patients operated for complicated cataract. We reported complete opacification of posterior capsular opening 7 months after first ND: YAG laser capsulotomy and opacification of posterior capsular opening in patients who had dehiscence in the posterior capsule during the surgical procedure. 

**Case-1**

A 70-year-old female patient had uneventful cataract surgery by phacoemulsification technique with implantation of in the bag hydrophilic intraocular lens (IOL) in her left eye.

**Fig. 1:**
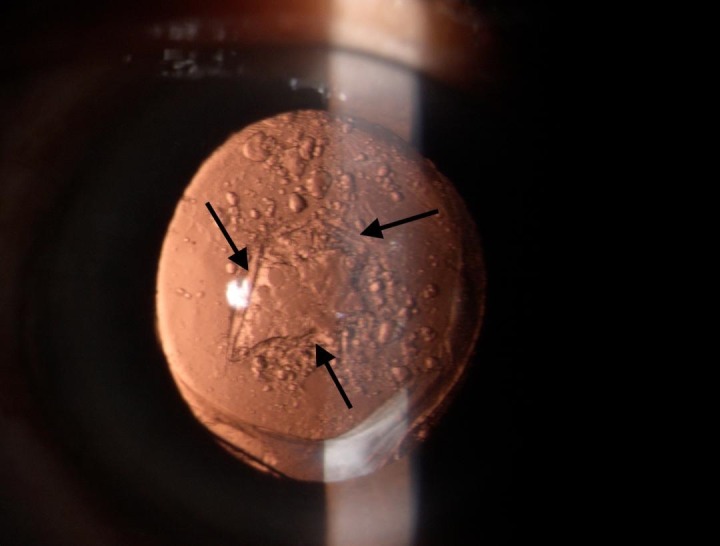
Arrows showing previous capsulotomy opening. The opening is occluded by the pearl like cells

Her postoperative visual recovery was excellent and the follow up was uneventful. Eight months after surgery, the patient developed posterior capsular opacification (PCO) for which ND: YAG capsulotomy was performed. Her pre-ND: YAG capsulotomy vision was 20/ 200, and improved to 20/ 20 post ND: YAG posterior capsulotomy. However, she returned after 7 months first post YAG capsulotomy with the blurring of vision and glare at night. Her visual acuity reduced to 20/ 200. Slit lamp examination revealed pearl like opacification of posterior capsule occupying the entire YAG capsulotomy opening (**Fig. 1**). 

The rest of the posterior capsule was showing pearl like opacification. Repeated ND: YAG capsulotomy restored her vision to 20/ 20. Fundus examination post YAG was normal. The follow up of the patient for one year did not reveal any recurrence. The right eye had an immature senile cataract. 

The patient was non-diabetic.

**Case-2**

**Fig. 2:**
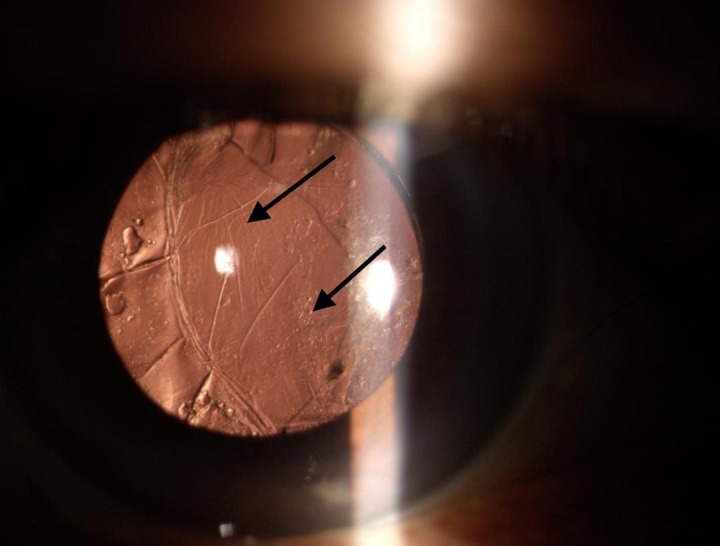
Arrows showing pearl and fibrotic posterior capsular opacification

A 60-year-old patient was operated for posterior polar cataract in his right eye. During the surgical procedure, intraoperative dehiscence of the posterior capsule occurred. No vitrectomy was needed as dehiscence happened at the end of the surgical procedure, after complete cortical cleanup and there was no vitreous loss. The patient was implanted with hydrophobic IOL in the capsular bag without performing an optic capture. Postoperative follow up was uneventful with good recovery of vision. There were no signs of inflammation on slit lamp examination on each visit. The patient presented 3 years later with occlusion of the posterior capsular opening with mixed pearl-like and fibrotic PCO (**Fig. 2**). 

The left eye had immature senile cataract with visual acuity of 20/ 60. The patient had non-insulin dependent diabetes for 10 years. Fundus examination showed non-proliferative diabetic retinopathy in both eyes. 

Both patients did not offer a history of suggestive postoperative inflammation.

## Discussion

Despite the developments in the cataract surgical procedure and IOL materials and design, posterior capsular opacification (PCO) remains a significant problem after cataract surgery. The only modality available for treatment is ND: YAG laser capsulotomy. Roger et al. have reported reopacification of visual axis after YAG capsulotomy in the tune of 0.7% [**2**]. 

We reported two cases with occlusion of posterior capsular opening with proliferating lens epithelial cells (LEC’s) in patients with ND: YAG capsulotomy for PCO. In the first case, complete occlusion of capsulotomy opening was observed 7 months after the YAG capsulotomy. Proliferating cells, which occluded capsulotomy opening, resembled epithelial pearl like cells. Jayaram et al. reported repeated ND: YAG capsulotomy in a series of five cases implanted with either PMMA or hydrogel lenses [**3**]. Kalliath et al. described a curious case of visual axis opacification in-patient implanted with hydrophobic acrylic IOL [**4**]. However, the patient had undergone vitrectomy with silicone oil injection for retinal detachment. In our case, hydrophilic IOL was implanted. The patient did not have any retinal and vitreous problem.

In the second case, opacification was not as severe as observed in the first case. Both pearl-like and fibrotic PCO was seen. Tassingnon et al. have shown reclosure of posterior capsulorhexis opening in eyes predisposed to postoperative inflammation [**5**]. Our patient had diabetes with non-proliferative diabetic retinopathy. Proliferation of LEC’s after ND: YAG capsulotomy has been reported in patients with proliferative vitreoretinal diseases including diabetic retinopathy [**6**]. Therefore, diabetes was ruled out as a cause for fibrotic and pearl-like PCO formation. 

**Fig. 3:**
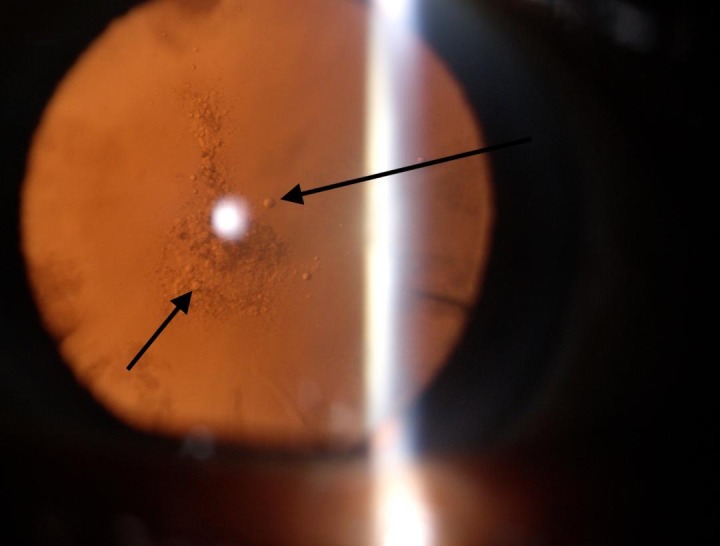
Posterior subcapsular cataract showing pearl like opacification

**Fig. 4:**
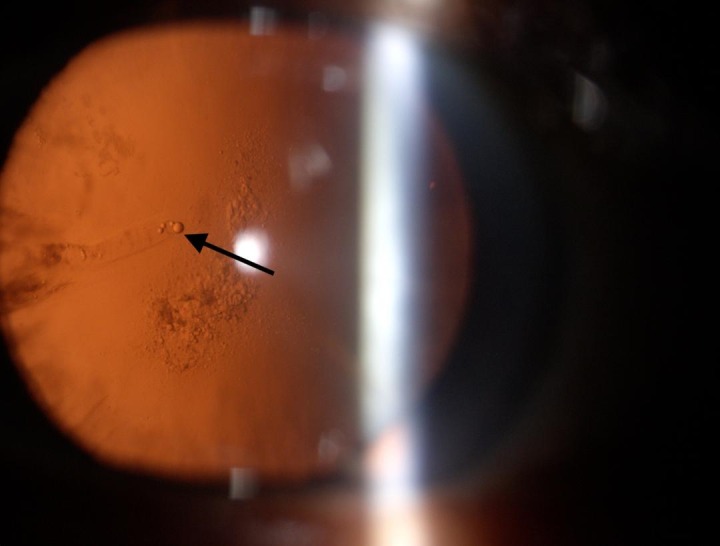
Cortical cataract showing pearl like cells

Both cases had pearl like opacification, which is believed to originate from the LEC’s [**7**]. This was substantiated by our observation of pearl-like cells seen in the posterior subcapsular cataract and cortical cataract (**Fig. 3**,**4**). These pearl-like cells were seen coming from the equatorial area of the lens and accumulating in the central part of the lens, causing cataractous changes in the visual axis. Similar types of cells were seen in the reopacified ND: YAG capsulotomy opening. 

Can there be any prevention to avoid proliferating LEC’s? One can have perfect 5-5.5mm capsulorhexis, which will cover the edge of IOL and extend beyond 0.5mm from the edge. This leads to the fusion of the anterior capsule with IOL. The proliferation of LEC’s to the posterior capsule can be avoided. 

These cases suggest reopacification of the posterior capsular opening, which is unrelated to the IOL material. In the first case, IOL implanted was of hydrophilic material and, in the second case, it was made up of hydrophobic material. 

In conclusion, pearl-like PCO is common to block the posterior capsular opening after ND: YAG capsulotomy. Repeated capsulotomy improves vision. Reopacification is unrelated to the IOL material.
